# Multicenter Study of the Risk Factors and Outcomes of Bloodstream Infections Caused by Carbapenem-Non-Susceptible *Acinetobacter baumannii* in Indonesia

**DOI:** 10.3390/tropicalmed7080161

**Published:** 2022-07-31

**Authors:** Dewi Anggraini, Dewi Santosaningsih, Pepy Dwi Endraswari, Novira Jasmin, Fajri Marindra Siregar, Usman Hadi, Kuntaman Kuntaman

**Affiliations:** 1 Doctoral Program of Medical Science, Faculty of Medicine, Universitas Airlangga, Surabaya 60115, Indonesia; dewi.anggraini-2019@fk.unair.ac.id; 2 Department of Microbiology, Faculty of Medicine, Universitas Riau, Pekanbaru 28133, Indonesia; novirajasminmd@gmail.com; 3 Arifin Achmad General Hospital, Pekanbaru 28111, Indonesia; fajrifkunri@gmail.com; 4 Department of Clinical Microbiology, Faculty of Medicine, Universitas Brawijaya, Malang 65145, Indonesia; dewi.santosa@ub.ac.id; 5 Department of Clinical Microbiology, Dr. Saiful Anwar Hospital, Malang 65112, Indonesia; 6 Department of Medical Microbiology, Faculty of Medicine, Universitas Airlangga, Surabaya 60115, Indonesia; pepy.dr@fk.unair.ac.id; 7 Department of Medical Microbiology, Dr. Soetomo General Academic Hospital, Surabaya 60286, Indonesia; 8 Department of Biochemistry, Faculty of Medicine, Universitas Riau, Pekanbaru 28133, Indonesia; 9 Department of Internal Medicine, Faculty of Medicine, Universitas Airlangga, Surabaya 60115, Indonesia; usmanhadi@sby.centrin.net.id; 10 Department of Internal Medicine, Dr. Soetomo General Academic Hospital, Surabaya 60286, Indonesia

**Keywords:** *Acinetobacter baumannii*, bacteremia, carbapenem resistance, infectious disease, risk factor

## Abstract

The prevalence of bacteremia caused by carbapenem-non-susceptible *Acinetobacter baumannii* (CNSAB) continues to increase, and it is associated with a high mortality rate. Early recognition of infection and mortality determinants risk factors is necessary for adequate antibiotic administration. We aimed to determine the risk factors and outcomes of CNSAB bacteremia in Indonesia. A multicenter case-control study was conducted in three referral hospitals in Indonesia. Data were collected retrospectively from January 2019 to December 2021. Cases were defined as patients with bacteremia where CNSAB was isolated from the blood, while the controls were patients with bacteremia caused by carbapenem-susceptible *A. baumannii* (CSAB). Risk factors for bacteremia and mortality associated with CNSAB bacteremia were determined using univariates analysis (chi-squared and Student’s *t*-test or Mann–Whitney test) and multivariate logistic regression analysis. A total of 144 bacteremia patients were included, of whom 72 patients were for each case and control group. The final model of multivariate regression analysis revealed that bacteremia source from the lower respiratory tract (adjusted odds ratio (aOR): 3.24; 95% CI: 1.58–6.63, *p* = 0.001) and the use of central venous catheter (aOR: 2.56; 95% CI: 1.27–5.18; *p* = 0.009) were independent risk factors for CNSAB bacteremia. Charlson Comorbidity Index ≥ 4 (aOR: 28.56; 95% CI: 3.06–265.90, *p* = 0.003) and Pitt Bacteremia Score ≥ 4 (aOR: 6.44; 95% CI: 1.17–35.38; *p* = 0.032) were independent risk factors for mortality due to CNSAB bacteremia. Only high Pitt Bacteremia Score was an independent risk factor for mortality of CSAB bacteremia. In conclusion, we identified the risk factors for CNSAB-associated bacteremia and the risk factors for death, which are relevant for empiric therapy and infection control prevention, as well as prognosis evaluation of patients with bloodstream infections.

## 1. Introduction

*Acinetobacter baumannii* (*A. baumannii*) is a Gram-negative coccobacillus that is a significant cause of hospital-associated infections worldwide, particularly lower respiratory tract infections and bacteremia [1,2]. *A. baumannii* can accumulate various antibiotic resistance mechanisms to create multidrug-resistant strains, including resistance to carbapenem antibiotics [1,3]. *A. baumannii* is a member of the ESKAPE pathogen, which is a group of pathogens responsible for the majority of nosocomial infections and are capable of escaping the biocidal action of antimicrobial agents [4]. Bacteremia caused by multidrug-resistant *A. baumannii* is associated with a high mortality rate [5,6,7,8,9,10,11]. According to a meta-analysis, the mortality rate from bacteremia caused by carbapenem-resistant *A. baumannii* also known as carbapenem-non-susceptible *A. baumannii* (CNSAB), is 33% [11]. The limited antibiotic therapeutic options for bacteremia caused by CNSAB make managing this infection challenging [12]. The prevalence of bacteremia caused by CNSAB is increasing globally, including in Indonesia [13]. According to the Indonesian National Surveillance on Antimicrobial Resistance data in 2020, 61% of *A. baumannii* isolates from blood specimens were confirmed as carbapenem-resistant [14].

Several studies have demonstrated that inappropriate empirical antibiotic therapy is one of the factors associated with increase mortality due to bacteremia caused by CNSAB [5,8,9,15,16,17]. Therefore, early recognition of risk factors of CNSAB bacteremia is necessary to provide adequate antibiotics. Previous studies have identified some of the risk factors associated with CNSAB bacteremia such as old age, male, history of previous use of antibiotics, history of previous chemotherapy or radiotherapy, history of colonization by *A. baumannii*, history of prolonged hospital stay, and use of invasive devices such as central venous catheters, ventilators, or drainage catheters [6,7,10,15,16,18,19,20,21,22,23,24]. However, the risk factors, and outcomes of CNSAB bacteremia in developing countries, including Indonesia, are limited. Since factors associated with clinical characteristics, risk factors, and outcomes of CNSAB bacteremia could be affected by a variety of factors, including ethnic, geographic, environmental, and economic factors, CNSAB prevalence, and differences in clinical practice and antibiotic prescribing habits [6], studies from developing countries are essential. Therefore, this study sought insights into CNSAB bacteremia from a developing country. The aims of this study were (1) to determine the risk factors for CNSAB bacteremia and (2) to determine the outcomes of CNSAB bacteremia and its associated determinants in Indonesia.

## 2. Methods

### 2.1. Study Design, Setting and Patients

A multicenter case-control study was conducted in three province referral hospitals located on two major islands in Indonesia: Arifin Achmad Hospital (600-bed hospital) in Pekanbaru (Sumatra Island); Dr. Soetomo Hospital (1500-bed hospital) in Surabaya (Java Island), and Dr. Saiful Anwar Hospital (800-bed hospital) in Malang (Java Island).

**Figure 1:**
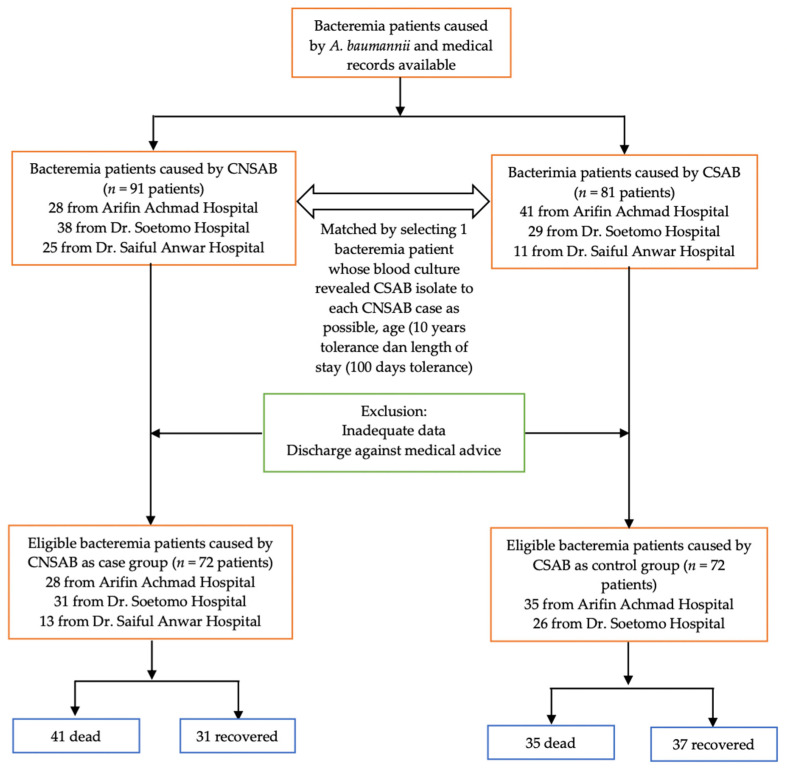
Flowchart of the sample selection.

Cases were defined as bacteremia patients from whom CNSAB was isolated from their blood. Controls were patients with bacteremia from whom carbapenem-susceptible *A. baumannii* (CSAB) was isolated from the blood specimen. Samples were collected retrospectively from January 2019 to December 2021, with the ratio of cases and controls being 1:1. Controls were matched with the cases based on the time of hospital admission (i.e., the control and case were hospitalized at approximately the same time), age, and length of hospital stay. Patients with the absence of medical records or inadequate data and discharged against medical advice were excluded. The flowchart of the enrolled cases and controls is described in Figure 1. A case report form (CRF) was prepared and used to collect the data from the medical records and the hospital databases.

### 2.2. Blood Culture and Antibiotic Resistance Test

The automated Bact/Alert (bioMérieux, Marcy l’Etoile, France) or automated BACTEC system (Becton Dickinson, Franklin Lakes, NJ, USA) were used to culture the blood samples. The automated Vitek 2 System (Biomerieux, Marcy l’Etoile, France) or Phoenix (BD, Franklin Lakes, NJ, USA) were used for identification and antibiotic resistance tests in all study sites. Vitek 2 System was used in Arifin Achmad Hospital and Dr. Saiful Anwar Hospital, whereas Dr. Soetomo Hospital used Phoenix for identification and susceptibility testing. The interpretation of antibiotic resistance tests followed the Clinical and Laboratory Standards Institute (CLSI) in all hospitals. To confirm the carbapenemase production of CNSAB isolates, a molecular examination to identify the carbapenemase genes was conducted. All of the tested isolates carried carbapenemase genes; the results have been published elsewhere [25].

### 2.3. Study Variables and Definitions

There were two response variables assessed in this study: (1) type of bacteremia based on the antibiotic resistance profile of *A. baumannii* (CNSAB bacteremia vs. CSAB bacteremia) and (2) the outcome of bacteremia caused by CNSAB (recovered vs. death). CNSAB was defined if the antibiotic sensitivity test showed resistance results to one of the carbapenem antibiotics (meropenem, imipenem, or doripenem), while CSAB was defined as samples with sensitive *A. baumannii* to all carbapenem antibiotics. The outcome of CNSAB bacteremia was classified as recovered or death. Death refers to hospital mortality.

Several risk factors for CNSAB bacteremia were collected in this study, including age, sex, type of ward, whether the patient was referred from another hospital, the length of onset and hospital stay, days to mortality, type or origin of the isolates, source of bacteremia, severity of sepsis, and comorbid factors. In addition, data on surgical history, antibiotic use history, immunosuppressant medication history within three months, and hospitalization history within the last 12 months were also collected.

The length of onset was the duration from the date of hospitalization to the date of bacteremia onset, and the length of stay was defined as the duration from the date of hospitalization to the date of hospital discharge. Days to mortality was the duration from the date of hospitalization to the date of death. Type of *A. baumannii* isolate was classified as hospital isolate if the date of onset of bacteremia occurred more than two days after hospital admission or if the patient was referred from another hospital where the patient had been treated for two days, while community isolate was defined if the date of bacteremia onset occurred ≤2 days after hospital admission [26].

Sources of bacteremia were grouped into primary and secondary. Primary bacteremia is not secondary to an infection at another body site. Secondary bacteremia could be an infection from the upper and lower respiratory tract, urinary tract, skin and soft tissue, reproduction, surgical wounds, gastrointestinal tract, and central nervous system. For statistical purposes, bacteremia originating from the lower respiratory tract, the dominant source, was also compared to bacteremia due to other sources.

The severity of sepsis was assessed using a Pitt Bacteremia Score as previously proposed [27]. The scores were calculated at the onset of bacteremia and included the indicator of temperature, blood pressure, consciousness, presence of cardiac arrest, and use of mechanical ventilation [27]. In addition, the level of consciousness (alert, stupor, disorientation, coma, or sedation) was assessed, and for the statistical purpose, it was divided into two groups: alert and disturbance (stupor, disorientation, coma, or sedation). The Pitt Bacteremia Score ranges from 0 to 14 points, with a score ≥ 4 commonly used as an indicator of critical illness and increased mortality rate [28].

The patient’s comorbid factor was calculated using the Charlson Comorbidity Index, which includes age, myocardial infarction, chronic heart failure, peripheral vascular disease, cerebrovascular accident, dementia, chronic obstructive pulmonary disease, connective tissue disease, peptic ulcer, hemiplegia, chronic kidney disease, leukemia, lymphoma, HIV/AIDS, solid tumors, diabetes mellitus, and liver disorders [29]. In this study, the Charlson Comorbidity Index was categorized into two groups: <4 and ≥4, based on the previous study [30].

### 2.4. Statistical Analysis

Demographic data, type of ward, whether the patient was referred from another hospital, length of onset, length of hospital stay, days to mortality, type of the isolates, source of bacteremia, the severity of sepsis, comorbid factors, surgical history, antibiotic use history, immunosuppressant medication history within three months, and hospitalization history were included in the risk factors analysis.

The univariate analyses (Chi-squared test and Student’s t-test or Mann–Whitney test) were performed based on the type of data to determine the risk factors associated with the bacteremia caused by CNSAB and determinants associated with CNSAB mortality. All variables with *p* ≤ 0.25 within the univariate analysis were included in the multivariate logistic regression analysis. The final multivariate analysis model used the backward elimination approach, in which variables that had the least significant effect on the model were removed. The crude odds ratio (OR) and adjusted OR (aOR) were calculated during univariate and multivariate, respectively, together with the 95% confidence intervals (95% CIs). The significance was assessed using *p* < 0.05. All statistical analyses were conducted using Stata version 16 software (College Station, TX: Stata Corp LLC, TX, USA).

## 3. Results

### 3.1. Risk Factors Associated with CNSAB Bacteremia

In this study, there was an equal number of bacteremia patients in the CNSAB (case group) and CSAB (control group): 72 patients for each group were included. There was no significant difference in the distribution of cases and controls based on site, island, and year of sampling (see Table S1 Supplementary files). The comparison of demographics and risk factors between CNSAB and CSAB bacteremia are presented in Table 1.

Univariate analysis revealed that intensive care unit (ICU) admission (OR: 2.35; 95% CI: 1.06–5.19), bacteremia source from the lower respiratory tract (OR: 3.49; 95% CI: 1.73–7.03), use of mechanical ventilation (OR 2.00; 95 CI: 1.02–3.92), having diabetes mellitus (OR: 3.94; 95% CI: 1.47–10.58), and use of central venous catheter (OR: 2.80; 95% CI: 1.42–5.52) were associated with the risk of CNSAB bacteremia (Table 1).

Multivariate logistic regression analysis was performed to assess the independent factors of CNSAB bacteremia by including variables with a *p* ≤ 0.25 in univariate analyses. The variables included in the multivariate analysis were sex, type of ward, bacteremia source, the presence of hypotension, use of mechanical ventilation, presence of diabetes mellitus, central venous catheter use, and the history of previous antibiotic use. The final model revealed that the source of bacteremia originating from the lower respiratory tract (aOR: 3.24; 95% CI: 1.58–6.63, *p* = 0.001) and the use of central venous catheter (aOR: 2.56; 95% CI: 1.27–518, *p* = 0.09) were the independent risk factors for bacteremia caused by CNSAB (Table 2).

### 3.2. Risk Factors Associated with the Mortality of CNSAB and CSAB Bacteremia

Our study analyzed the mortality risk factors of both CNSAB and CSAB bacteremia. The risk factors were slightly different between CNSAB and CSAB bacteremia. In CNSAB bacteremia, univariate analysis found that older age (*p* = 0.018), bacteremia source from the lower respiratory tract (OR: 3.07; 95% CI: 1.03–9.11), high Pitt Bacteremia Score (*p* < 0.001), Pitt Bacteremia Score ≥ 4 (OR: 16.18; 95% CI: 4.20–62.38), hypotension (OR: 5.97; 95% CI: 1.56–22.95), cardiac arrest (*p* = 0.009), consciousness disturbance (OR: 14.54; 95% CI: 4.21–50.19), high Charlson Comorbidity Index (*p* < 0.001), or Charlson Comorbidity Index ≥ 4 (OR: 9.28; 95% CI: 1.94–44.35) were associated with death (Table 3).

In CSAB bacteremia, the risk factors of mortality were being admitted to the ICU (OR: 3.05; 95% CI: 1.06–8.74), high Pitt Bacteremia Score (*p* < 0.001), Pitt Bacteremia Score ≥ 4 (OR: 16.00; 95% CI: 4.85–52.82), fever (OR: 3.34; 95% CI: 1.03–10.79), hypotension (OR: 7.00; 95% CI: 1.41–34.76), using a mechanical ventilator (OR: 5.91; 95% CI: 2.14–16.34), cardiac arrest (*p* < 0.001), having a disturbance of consciousness (OR: 14.46; 95% CI: 4.63–45.22), using central venous catheter (OR: 4.05; 95% CI: 1.41–11.64), and having a history of using antibiotics previously (OR: 3.54; 95% CI: 1.34–9.34) (Table 4).

Multivariate logistic regression analysis was carried out to assess the independent determinants of death due to CNSAB and CSAB bacteremia by including variables with a *p* < 0.25 in univariate analysis. In the final model, two independent risk factors for mortality of bacteremia due to CNSAB were identified: the Pitt Bacteremia Score ≥ 4 (aOR: 13.29; 95% CI: 3.31–53.33, *p* < 0.001) and Charlson Comorbidity Index ≥ 4 (aOR: 6.44; 95% CI: 1.17–35.38, *p* = 0.032). Meanwhile, in CSAB bacteremia, only one independent factor associated with death, which was having a high Pitt Bacteremia Score (aOR: 1.87; 95% CI: 1.41–2.47, *p* < 0.001) (Table 5).

The patients with CNSAB bacteremia with the Pitt Bacteremia Score ≥ 4 were at approximately 13.29 times higher risk of death than patients with index less than 4; aOR: 13.29; 95% CI: 3.31–53.33 with *p* < 0.001. In addition, those with the Charlson Comorbidity Index ≥4 had higher odds of mortality than those with a score less than 4 with OR: 6.44; 95% CI: 1.17–35.38 and *p* = 0.032 (Table 5).

## 4. Discussion

To the best of our knowledge, this is the first multicenter case-control study in Indonesia to investigate the risk factors for CNSAB bacteremia and determinants of CNSAB bacteremia mortality. Previous studies have identified several risk factors related to bacteremia caused by multidrug-resistant *A. baumannii*, including CNSAB [6,7,10,15,16,18,19,20,21,22,23,24]. In the present study, ICU admission, infection source of lower respiratory tract infection, ventilator or central venous catheter use, and the presence of diabetes mellitus were found to be more common in bacteremia caused by CNSAB than CSAB. Patients admitted to the ICU are in severe condition, have already been exposed to multiple antibiotics, and have a compromised immune system. This condition makes the patients are susceptible to cross-transmission infection from health workers or other patients and invasive procedures after a lengthy hospital stay [9,10,12,14,16].

*A. baumannii* is able to survive in the environment and medical equipment for long period. Bacteremia occurs when the bacteria entering the patient’s exposed skin or mucosal barriers during invasive procedures [10]. The use of mechanical ventilator and central venous catheter indicates that the patient is in severe condition and is at risk of suffering from ventilator-associated pneumonia and central line-associated bloodstream infection, which are commonly caused by multidrug-resistant bacteria, including CNSAB [5,6,7,12,15,23]. Using ventilators and central venous catheters, both risk factors for bacteremia caused by CNSAB, emphasizes the importance of strictly adhering to the ventilator and central venous catheter bundle in the ICU [31,32,33].

One of the clinical manifestations of *A. baumannii* infection is lower respiratory tract infection [17]. In our study, the most frequent source of infection from bacteremia due to *A. baumannii* was lower respiratory tract infection. Interestingly, the lower respiratory tract infection was more frequently the source of bacteremia due to CNSAB than CSAB and was an independent risk factor in multivariate analysis. Similar findings also have been reported previously [10,17]. These could provide insight to medical practitioners to be more aware of the possible CNSAB bacteremia among patients with lower respiratory tract infections.

In the present study, diabetes was found more common in bacteremia caused by CNSAB than CSAB. Diabetes is a significant public health concern globally, and Indonesia is the country with the fifth-most prevalent diabetes among individuals, with 19.5 million cases [34]. A study in Saudi Arabia found that *A. baumannii* isolated from diabetic patients harbored a significantly higher frequency to have carbapenem resistance [21]. Many studies have indicated that diabetic patients have a higher risk of antibiotic resistance than non-diabetic patients [18,19,20]. Due to diabetic-associated complications and decreased immunity, diabetic patients are more susceptible to repeated bacterial infections and antibiotic exposure [20], particularly in countries with relatively less tight antibiotic use, such as Indonesia. These could cause infection-associated bacteria in diabetic patients to be more resistant, including *A. baumannii* [20]. Therefore, blood sugar monitoring, diagnosis, and prompt and aggressive antibiotic therapy are critically needed in diabetic patients with infections, in line with the implementation of national antibiotic usage policies to reduce antibiotic resistance rates [20].

The prognosis of bacteremia patients caused by CNSAB depends on the severity of the disease and the patient’s immunological status. Previous studies found that high Pitt Bacteremia Score and Acute Physiology and Chronic Health Evaluation II (APACHE II), the presence of septic shock, multi-organ failure, pneumonia, ICU admission, use of ventilator and central venous catheter, increased leukocyte count, and low albumin levels were all associated with disease severity of CNSAB bacteremia [2,5,8,9,10,11,12,14,17]. Meanwhile, the patient’s immunological status factor included old age, the presence of comorbid factors such as liver disease, chronic kidney disease, hypertension, diabetes, malignancy, post-transplantation, and receiving immunosuppressant therapy [2,11,14,23]. In our study, some of those mentioned factors were more common in patients dying of CNSAB bacteremia; high Pitt Bacteremia Score and high Charlson Comorbidity Index (score ≥ 4) were identified as independent risk factors for mortality of CNSAB bacteremia in the final model. Pitt Bacteremia Score is easy to use. In addition, studies found that it could predict the clinical outcomes in bloodstream infections due to *Pseudomonas aeruginosa, Enterobacter, Klebsiella,* and multidrug-resistant *A. baumannii* [2,14]. These data suggest that the comorbidities of the patients with CNSAB infection must be assessed during infection to predict mortality risk. One simple way to assess the patient’s comorbidities is by using the Charlson Comorbidity Index, an instrument to measure the burden of comorbidity consisting of 19 categories related to chronic health problems [35]. Its calculation is only based on anamnesis, making it is relatively rapid, simple, and inexpensive.

### Study Limitations

There are some limitations of this study that need to be discussed. The matching tolerance for the length of stay between case and control was quite large (up to 100 days) due to the limited number of eligible controls. This study did not assess the antibiotic therapy of the patients, either empirical or definitive, as a factor influencing the mortality of the patients. Several studies have demonstrated that inappropriate empiric antibiotic therapy is an independent factor influencing mortality of CNSAB bacteremia [2,8,11,16,23,24]. The history of antibiotic use was also not differentiated by class of antibiotics in the present study because the information on antibiotic types is unavailable. Studies have revealed that using meropenem was the most prevalent history of antibiotic use associated with the incidence of bacteremia caused by multidrug-resistant *A. baumannii* [9,12,13,14,15,17,23]. Furthermore, piperacillin/tazobactam, cefoperazone/sulbactam, fourth-generation cephalosporins, aminoglycosides, linezolid, and colistin are risk factors for multidrug-resistant *A. baumannii* [9,13,16,17,23]. Therefore, a prospective study assessing the association between the type of antibiotic use and the mortality of CNSAB bacteremia needs to be conducted in the near future.

## 5. Conclusions

Our data suggest that the infection source of lower respiratory tract infections and central venous catheter use are independent risk factors for CNSAB bacteremia. High Pitt Bacteremia Score and Charlson Comorbidity Index (score ≥ 4) became independent determinants for death due to bacteremia caused by CNSAB. The determinants are slightly different in CSAB bacteremia, of which only Pitt Bacteremia Score ≥ 4 was the independent risk factor for mortality. Understanding these risk variables is essential for determining empiric therapy and infection control preventive priorities and assessing the patient’s prognosis.

## Figures and Tables

**Table 1:** Characteristics of respondents and potential risk factors for bacteremia due to CNSAB and CSAB (*n* = 144).

Variable	Bacteremia		Total	OR (95% CI)	*p*-Value
	CNSAB (*n* = 72) *n* (%)	CSAB (*n* = 72) *n* (%)			
Sex				1.59 (0.81–3.11)	0.170
Male	39 (54.2)	47 (65.3)	86 (59.7)		
Female	33 (45.8)	25 (34.7)	58 (40.3)		
Age, median (IQR) ^a^	41 (1–57)	40 (4–56)	40 (3–56)		0.810
Ward				2.35 (1.06–5.19)	0.033 *
Intensive care unit	60 (83.3)	49 (68.1)	109 (75.7)		
Non-intensive care unit	12 (16.7)	23 (31.9)	35 (24.3)		
Referred from another hospital	40 (55.6)	35 (48.6)	75 (52.1)	1.32 (0.69–2.55)	0.400
Origin of isolate					
Hospital	53 (73.6)	57 (79.2)	110 (76.4)	0.73 (0.34–1.59)	0.430
Community	19 (26.4)	15 (20.8)	34 (23.6)		
Length of onset, median (IQR) ^a^	6 (3–12)	6 (3–10)	6 (3–11)		0.980
Length of stay, median (IQR) ^a^	17 (9–28)	13 (7–23)	14 (8–26)		0.150
Days to mortality, median (IQR) ^a^	4 (2–8)	2 (1–8)	3 (1–8)		0.150
Outcome				1.39 (0.72–2.69)	0.320
Recovered	31 (43.1)	37 (51.4)	68 (47.2)		
Died	41 (56.9)	35 (48.6)	76 (52.8)		
Bacteremia source				3.18 (0.62–16.33)	0.220
Primary	2 (2.8)	6 (8.3)	8 (5.6)		
Secondary	70 (97.2)	66 (97.1)	136 (94.5)		
Bacteremia source				3.49 (1.73–7.03)	<0.001 **
Primary or another secondary	19 (26.4)	40 (55.6)	59 (41.0)		
Secondary-lower respiratory tract	53 (73.6)	32 (44.4)	85 (59.0)		
Pitt Bacteremia Score, median (IQR) ^a^	2 (2–6)	2 (0–6)	2 (0–6)		0.390
Pitt Bacteremia Score ≥ 4	29 (40.3)	30 (41.7)	59 (41.0)	0.94 (0.49–1.84)	0.870
Fever	13 (18.1)	17 (23.6)	30 (20.8)	0.71 (0.32–1.60)	0.410
Hypotension	19 (26.4)	12 (16.7)	31 (21.5)	1.79 (0.80–4.04)	0.160
Use of mechanical ventilator	48 (66.7)	36 (50.0)	84 (58.3)	2.00 (1.02–3.92)	0.043 *
Cardiac arrest	8 (11.1)	9 (12.5)	17 (11.8)	0.88 (0.32–2.41)	0.800
State of consciousness				0.89 (0.46–1.72)	0.740
Alert	40 (55.6)	38 (52.8)	78 (54.2)		
Disturbance	32 (44.4)	34 (47.2)	66 (45.8)		
Charlson Comorbidity Index, median (IQR) ^a^	1 (0–3)	1 (0–2)	1 (0–2)		0.740
Charlson Comorbidity Index ≥ 4	18 (25.0)	15 (20.8)	33 (22.9)	1.27 (0.58–2.76)	0.550
Diabetes mellitus	19 (26.4)	6 (8.3)	25 (17.4)	3.94 (1.47–10.58)	0.004 *
Use of central venous catheter	42 (58.3)	24 (33.3)	66 (45.8)	2.80 (1.42–5.52)	0.003 *
Previous use of antibiotics history	45 (62.5)	36 (50.0)	81 (56.3)	1.67 (0.86–3.24)	0.130
Surgical history	34 (47.2)	36 (50.0)	70 (48.6)	0.90 (0.47–1.72)	0.740
Immunosuppressant therapy history	4 (5.6)	6 (8.3)	10 (6.9)	0.65 (0.18–2.40)	0.510
Hospital treatment history	34 (47.2)	37 (51.4)	71 (49.3)	0.85 (0.44–1.63)	0.620

CI: confidence interval, CNSAB: carbapenem-non-susceptible *A. baumannii*, CSAB: carbapenem susceptible *A. baumannii,* NA: not applicable, OR: odds ratio, IQR: interquartile range. * Significant at *p* < 0.05. ** Significant at *p* < 0.001. ^a^ Analyzed using Mann–Whitney test.

**Table 2:** Final model of multivariate analysis showing the risk factors of bacteremia due to CNSAB (*n* = 144).

Variable	aOR	95% CI	*p*-Value
Source of infection-lower respiratory tract	3.24	1.58–6.63	0.001 *
Use of central venous catheter	2.56	1.27–5.18	0.009 *

CI: confidence interval, aOR: adjusted odds ratio. * Significant at *p* < 0.05.

**Table 3:** Risk factors for mortality of bacteremia caused by CNSAB (*n* = 72).

Variable	Death (*n* = 41) *n* (%)	Recovered (*n* = 31) *n* (%)	Total (*n* = 72) *n* (%)	OR (95% CI)	*p*-Value
Sex				0.60 (0.23–1.55)	0.290
Male	20 (48.8)	19 (61.3)	39 (54.2)		
Female	21 (51.2	12 (38.7)	33 (45.8)		
Age, median (IQR) ^a^	48 (22–59)	18 (0–47)	41 (1–57)		0.018 *
Ward				0.61 (0.17–2.25)	0.460
Intensive care unit	33 (80.5)	27 (87.1)	60 (83.3)		
Non-intensive care unit	8 (20.0)	4 (12.9)	12 (16.7)		
Days to mortality, median (IQR) ^a^	4 (2–8)		4 (2–8)		NA
Other hospital referrals, median (IQR) ^a^	24 (58.5)	16 (51.6)	40 (55.6)	1.32 (0.52–3.39)	0.560
Origin of isolate					
Hospital	32 (78.0)	21 (67.7)	53 (73.6)	1.69 (0.59–4.87)	0.330
Community	9 (22.0)	10 (32.3)	19 (26.4)		
Bacteremia source				NA	0.210
Primary	2 (4.9)	0 (0.0)	2 (2.8)		
Secondary	34 (95.1)	31 (100.0)	70 (97.2)		
Bacteremia source				3.07(1.03–9.11)	0.039 *
Primary + another secondary	7 (17.1)	12 (38.7)	19 (26.1)		
Secondary-lower respiratory tract	34 (82.9)	19 (61.3)	53 (73.6)		
Pitt Bacteremia score, median (IQR) ^a^	4 (2–6)	2 (0–2)	2 (2–6)		<0.001 **
Pitt Bacteremia Score ≥ 4	26 (63.4)	3 (9.7)	29 (40.3)	16.18 (4.20–62.38)	<0.001 **
Fever	9 (22.0)	4 (12.9)	13 (18.1)	1.90 (0.53–6.86)	0.320
Hypotension	16 (39.0)	3 (9.7)	19 (26.4)	5.97 (1.56–22.95)	0.005 *
Use of mechanical ventilator	28 (68.3)	21 (67.7)	49 (68.1)	0.92 (0.34–2.48)	0.960
Cardiac arrest	8 (19.5)	0 (0.0)	8 (11.1)	NA	0.009 *
State of consciousness				14.54 (4.2–50.19)	<0.001 **
Alert	13 (31.7)	27 (87.1)	40 (55.6)		
Disturbance	28 (68.3)	4 (12.9)	32 (44.4)		
Charlson Comorbidity Index, median (IQR) ^a^	2 (0–4)	0 (0–2)	1 (0–3)		<0.001 **
Charlson Comorbidity Index ≥ 4	16 (39.0)	2 (6.5)	18 (25.0)	9.28 (1.94–44.35)	0.002 *
Diabetes mellitus	12 (29.3)	7 (22.6)	19 (26.4)	1.41 (0.48–4.17)	0.520
Liver disorder	1 (2.4)	0 (0.0)	1 (1.4)	NA	0.380
Use of central venous catheter	27 (65.9)	15 (48.4)	42 (58.3)	2.06 (0.79–5.35)	0.140
Previous use of antibiotics history	28 (68.3)	17 (54.8)	45 (62.5)	1.77 (0.68–4.66)	0.240
Surgical history	21 (51.2)	13 (41.9)	34 (47.2)	1.45 (0.57–3.72)	0.430
Immunosuppressant therapy history	3 (7.3)	1 (3.2)	4 (5.6)	2.37 (0.23–23.94)	0.450
Hospital treatment history	21 (51.2)	13 (41.9)	34 (47.2)	1.45 (0.57–3.72)	0.430

CI: confidence interval, CNSAB: carbapenem non-susceptible *A. baumannii*, NA: not applicable, OR: odds ratio, IQR: interquartile range. * Significant at *p* < 0.05.** Significant at *p* < 0.001. ^a^ Analyzed using Mann–Whitney test.

**Table 4:** Risk factors for mortality of bacteremia caused by CSAB (*n* = 72).

Variable	Death (*n* = 35) *n* (%)	Recovered (*n* = 37) *n* (%)	Total (*n* = 72) *n* (%)	OR (95% CI)	*p*-Value
Sex				0.81 (0.31–2.15)	0.670
Male	22 (63%)	25 (68%)	47 (65%)		
Female	13 (37%)	12 (32%)	25 (35%)		
Age, median (IQR) ^a^	51 (8–59)	34 (4–45)	40 (4–56)		0.065
Ward				3.05 (1.06–8.74)	0.035 *
Intensive care unit	28 (80%)	21 (57%)	49 (68%)		
Non-intensive care unit	7 (20%)	16 (43%)	23 (32%)		
Days to mortality, median (IQR) ^a^	2 (1–8)		2 (1–8)		
Other hospital referrals, median (IQR) ^a^	15 (43%)	20 (54%)	35 (49%)	0.64 (0.21–1.62)	0.340
Origin of isolate				3.28 (0.93–11.53)	0.056
Hospital	31 (89%)	26 (70%)	57 (79%)		
Community	4 (11%)	11 (30%)	15 (21%)		
Bacteremia source				0.50 (0.09–2.92)	0.430
Primary	2 (6%)	4 (11%)	6 (8%)		
Secondary	33 (94%)	33 (89%)	66 (92%)		
Bacteremia source				1.39 (0.55–3.52)	0.490
Primary + Other secondary	17 (49%)	15 (41%)	32 (44%)		
Secondary-lower respiratory tract	18 (51%)	22 (59%)	40 (56%)		
Pitt Bacteremia score, median (IQR) ^a^	5 (3–8)	0 (0–2)	2 (0–6)		<0.001 **
Pitt Bacteremia Score ≥ 4	25 (71%)	5 (14%)	30 (42%)	16.00 (4.85–52.82)	<0.001 **
Fever	12 (34%)	5 (14%)	17 (24%)	3.34 (1.03–10.79)	0.038 *
Hypotension	10 (29%)	2 (5%)	12 (17%)	7.00 (1.41–34.76)	0.008 *
Use of mechanical ventilator	25 (71%)	11 (30%)	36 (50%)	5.91 (2.14–16.34)	<0.001 **
Cardiac arrest	9 (26%)	0 (0%)	9 (13%)	NA	<0.001 **
State of consciousness				14.46 (4.63–45.22)	<0.001 **
Alert	27 (77%)	7 (19%)	34 (47%)		
Disturbance	8 (23%)	30 (81%)	38 (53%)		
Charlson Comorbidity Index, median (IQR) ^a^	1 (0–3)	0 (0–2)	1 (0–2)		0.057
Charlson Comorbidity Index ≥ 4	9 (26%)	6 (16%)	15 (21%)	1.79 (0.56–5.69)	0.320
Diabetes mellitus	4 (11%)	2 (5%)	6 (8%)	2.26 (0.39–13.19)	0.360
Use of central venous catheter	17 (49%)	7 (19%)	24 (33%)	4.05 (1.41–11.64)	0.008 *
Previous use of antibiotics history	23 (66%)	13 (35%)	36 (50%)	3.54 (1.34–9.34)	0.009 *
Surgical history	17 (49%)	19 (51%)	36 (50%)	0.90 (0.36–2.26)	0.810
Immunosuppressant therapy history	2 (6%)	4 (11%)	6 (8%)	0.50 (0.09–2.92)	0.430
Hospital treatment history	21 (60%)	16 (43%)	37 (51%)	1.97 (0.77–5.03)	0.160

CI: confidence interval, CSAB: carbapenem susceptible *A. baumannii,* NA: not applicable, OR: odds ratio, IQR: interquartile range. * Significant at *p* < 0.05. ** Significant at *p* < 0.001. ^a^ Analyzed using Mann–Whitney test.

**Table 5:** The final model of multivariate analysis showing determinant of death of bacteremia due to CNSAB (*n* = 41) and CSAB (*n* = 35).

Variable	aOR	95% CI	*p*-Value
CNSAB			
Pitt Bacteremia Score ≥ 4	13.29	3.31–53.33	<0.001 **
Charlson Comorbidity Index ≥ 4	6.44	1.17–35.38	0.032 *
CSAB			
Pitt Bacteremia Score	1.87	1.41–2.47	<0.001 **

CI: confidence interval, CNSAB: carbapenem non-susceptible *A. baumannii*, CSAB: carbapenem susceptible *A. baumannii*, aOR: adjusted odds ratio. * Significant at *p* < 0.05. ** Significant at *p* < 0.001.

## Data Availability

The underlying data of this study are available from the corresponding author on request.

## Supplementary Materials

The following supporting information can be downloaded at: https://www.mdpi.com/article/10.3390/tropicalmed7080161/s1, Table S1: Comparison of case distribution of carbapenem susceptible *Acinetobacter baumannii* (CSAB) and carbapenem non-susceptible *Acinetobacter baumannii* (CNSAB) based on site, island, and year of sampling.
