# Supplementary material for: Multicenter Study of the Risk Factors and Outcomes of Bloodstream Infections Caused by Carbapenem-Non-Susceptible Acinetobacter baumannii in Indonesia
Source: Trop Med Infect Dis. 2022 Jul 31;7(8):161. doi: 10.3390/tropicalmed7080161 (PMC9412432; doi:10.3390/tropicalmed7080161)
Supplement: Supplementary file 1 [file tropicalmed-07-00161-s001.zip › tropicalmed-1812709-supplementary.pdf]

Supplementary files

Table S1. Comparison of case distribution of carbapenem susceptible *Acinetobacter baumannii* (CSAB) and carbapenem non-susceptible *Acinetobacter baumannii* (CNSAB) based on site, island, and year of sampling

	**CSAB N=72**	**CNSAB N=72**	**Total N=144**	**p-value**
Site				0.50
Arifin Achmad Hospital	35 (48.6%)	28 (38.9%)	63 (43.8%)	
Dr Soetomo Hospital	26 (36.1%)	31 (43.1%)	57 (39.6%)	
Dr Syaiful Anwar Hospital	11 (15.3%)	13 (18.1%)	24 (16.7%)	
Island				0.24
Sumatra	35 (48.6%)	28 (38.9%)	63 (43.8%)	
Java	37 (51.4%)	44 (61.1%)	81 (56.3%)	
Year				0.37
2019	16 (22.2%)	17 (23.6%)	33 (22.9%)	
2020	36 (50.0%)	42 (58.3%)	78 (54.2%)	
2021	20 (27.8%)	13 (18.1%)	33 (22.9%)	
